# iGC—an integrated analysis package of gene expression and copy number alteration

**DOI:** 10.1186/s12859-016-1438-2

**Published:** 2017-01-14

**Authors:** Yi-Pin Lai, Liang-Bo Wang, Wei-An Wang, Liang-Chuan Lai, Mong-Hsun Tsai, Tzu-Pin Lu, Eric Y. Chuang

**Affiliations:** 1 Bioinformatics and Biostatistics Core, Center of Genomic Medicine, National Taiwan University, Taipei, Taiwan; 2 Graduate Institute of Biomedical Electronics and Bioinformatics, Department of Electrical Engineering, National Taiwan University, Taipei, Taiwan; 3 Graduate Institute of Physiology, National Taiwan University, Taipei, Taiwan; 4 Institute of Biotechnology, National Taiwan University, Taipei, Taiwan; 5 Department of Public Health, Institute of Epidemiology and Preventive Medicine, National Taiwan University, Taipei, Taiwan

**Keywords:** Copy number alteration, Gene expression, R/Bioconductor

## Abstract

**Background:**

With the advancement in high-throughput technologies, researchers can simultaneously investigate gene expression and copy number alteration (CNA) data from individual patients at a lower cost. Traditional analysis methods analyze each type of data individually and integrate their results using Venn diagrams. Challenges arise, however, when the results are irreproducible and inconsistent across multiple platforms. To address these issues, one possible approach is to concurrently analyze both gene expression profiling and CNAs in the same individual.

**Results:**

We have developed an open-source R/Bioconductor package (iGC). Multiple input formats are supported and users can define their own criteria for identifying differentially expressed genes driven by CNAs. The analysis of two real microarray datasets demonstrated that the CNA-driven genes identified by the iGC package showed significantly higher Pearson correlation coefficients with their gene expression levels and copy numbers than those genes located in a genomic region with CNA. Compared with the Venn diagram approach, the iGC package showed better performance.

**Conclusion:**

The iGC package is effective and useful for identifying CNA-driven genes. By simultaneously considering both comparative genomic and transcriptomic data, it can provide better understanding of biological and medical questions. The iGC package’s source code and manual are freely available at https://www.bioconductor.org/packages/release/bioc/html/iGC.html.

**Electronic supplementary material:**

The online version of this article (doi:10.1186/s12859-016-1438-2) contains supplementary material, which is available to authorized users.

## Background

Genomic and transcriptomic data obtained from high-throughput technologies, such as microarray or next-generation sequencing have been widely utilized to elucidate the etiology and molecular mechanisms of multiple diseases [1, 2]. Genome-wide gene expression (GE) analysis can not only help to reveal the pathogenic process in a disease [3, 4] but also identify diagnostic and predictive biomarkers [5, 6]. However, the low reproducibility of identified biomarkers poses a major challenge in translating them into practical applications. One possible strategy to increase the reproducibility is to perform an integrated analysis of GE and copy number alteration (CNA; also called copy number variation) [7–10]. Previous studies have demonstrated that it is essential to identify prognostic biomarkers in independent datasets [11, 12]. The most popular method for integrating GE and CNA data from independent sources is to use a Venn diagram [12–15]. In this method, gene sets showing significant changes in GE are overlapped with gene sets showing significant changes in CNA. The Venn diagram method has two major drawbacks. First, because significant changes in GE and CNA are identified in the two platforms separately, their union does not guarantee that the changes happen simultaneously in the same patient. Therefore, the changes in GE are not directly driven by CNAs, which thwarts the purpose of the integrated analysis. Second, the union set of genes is usually not robust, to the extent that even a small change in a parameter may lead to dramatically different gene pools. To address these issues, we developed a new package to identify differentially expressed genes driven by CNAs from samples with both GE and CNA data. That is, for each gene, the samples are classified into different groups based on their CNA status, and Student’s *t*-test with unequal variance is then performed on the GE level. The results of the analyses of two real datasets and one published study demonstrated that the proposed approach is able to identify CNA-driven differentially expressed genes [16].

## Implementation

**Fig. 1:**
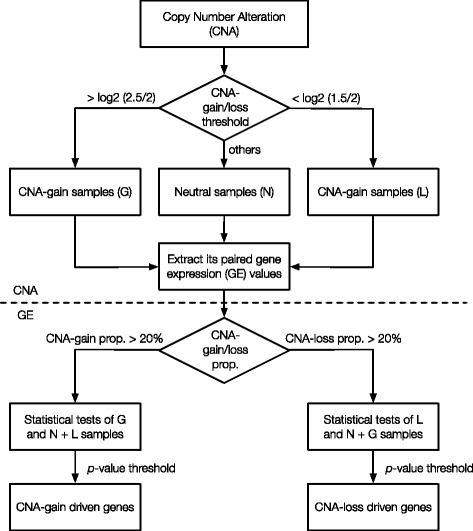
The overall workflow of iGC. Two parameters are defined: the minimum CN changes to classify samples as G or L groups, and the minimum sample proportion showing CNAs in a population

In order to perform an integrated analysis of GE and CNA (iGC), we developed a new package written in R. The overall flowchart is summarized in Fig. 1. Initially, for each gene, the samples are divided into three groups based on CNA status: CNA-gain (G), CNA-loss (L) and neutral (N), meaning no change in copy number. For a gene to be classified as G or L, the ratio of the number of samples with CNAs to the total number of samples must be larger than a given threshold. Lastly, statistical tests are performed at the GE level (G versus L + N groups or L versus G + N groups) based on whether the CN of the gene of interest is increased or decreased.

Briefly, input data can be directly imported from The Cancer Genome Atlas (TCGA) [17] and the Gene Expression Omnibus (GEO) [18]. Notably, all GE and CN data from different individuals must be normalized to the common baselines before performing the analysis with the iGC package. Multiple data formats are supported by specifying custom reader functions. Initially, input CN segments are mapped to the human genome and a threshold is given to define CNA-gain and CNA-loss (default values are set as 2.5 for gain and 1.5 for loss). To focus on dysregulated genes in the general population, only genes showing CNAs in at least 20% of the samples will be analyzed further. This threshold can be changed by the user. For the remaining genes, their GE levels are evaluated by Student’s *t*-test with unequal variance. False discovery rate, *p*-value and associated statistics are summarized in output files. The iGC package can accept gene expression data from different experimental platforms as long as the basic assumptions of Student’s *t*-test are not violated. Gene set enrichment analysis can be directly performed on the output files [19]. More details and examples can be found in the additional files.

### Simulation study and performance comparison with the SIM [20] package

To compare the performance of iGC and SIM, a set of simulated CN and GE data was analyzed by both packages concurrently. The mvtnorm package in R was utilized to generate simulated data. Previous studies have indicated the frequencies of CNA in the human genome can range from 5–50% [16, 21], and thus we set the CNA frequency of the simulated data to 30%. Furthermore, a study in breast cancer has demonstrated that only approximately 12% of the GE changes can be explained by their associated CNAs [22]. Therefore, the parameters for the simulation study were set as follows. The CN of a gene with CNA follows the normal distribution ~ N (3,0.2), whereas the CN of a gene without CNA follows the normal distribution ~ N (2,0.2). The GE levels of a gene with CNA follow the distribution ~ N (5,0.2), whereas the expression of a gene without CNA follows the distribution ~ N (2.5,0.2). Four conditions of the Pearson correlation between GE and CN were simulated to mimic the different levels of correlation. The Pearson correlations for the four conditions were 0.7–1, 0.3–0.7, 0–0.3 and 0 and each condition contains the same number of genes. To evaluate the consistency, two numbers of genes were tested: 100 and 300. Thus, each condition has 25 and 75 genes while the total number of genes is 100 or 300, respectively. We defined the genes with the highest correlation (*r* = 0.7–1) as true positive data and the other three conditions as true negative data. Four sample sizes were simulated to mimic different numbers of patients are analyzed: 50, 100, 200 and 300. One thousand simulations were run in each package for each combination of sample size and gene number.

## Results and Discussion

### Simulation study

**Fig. 2:**
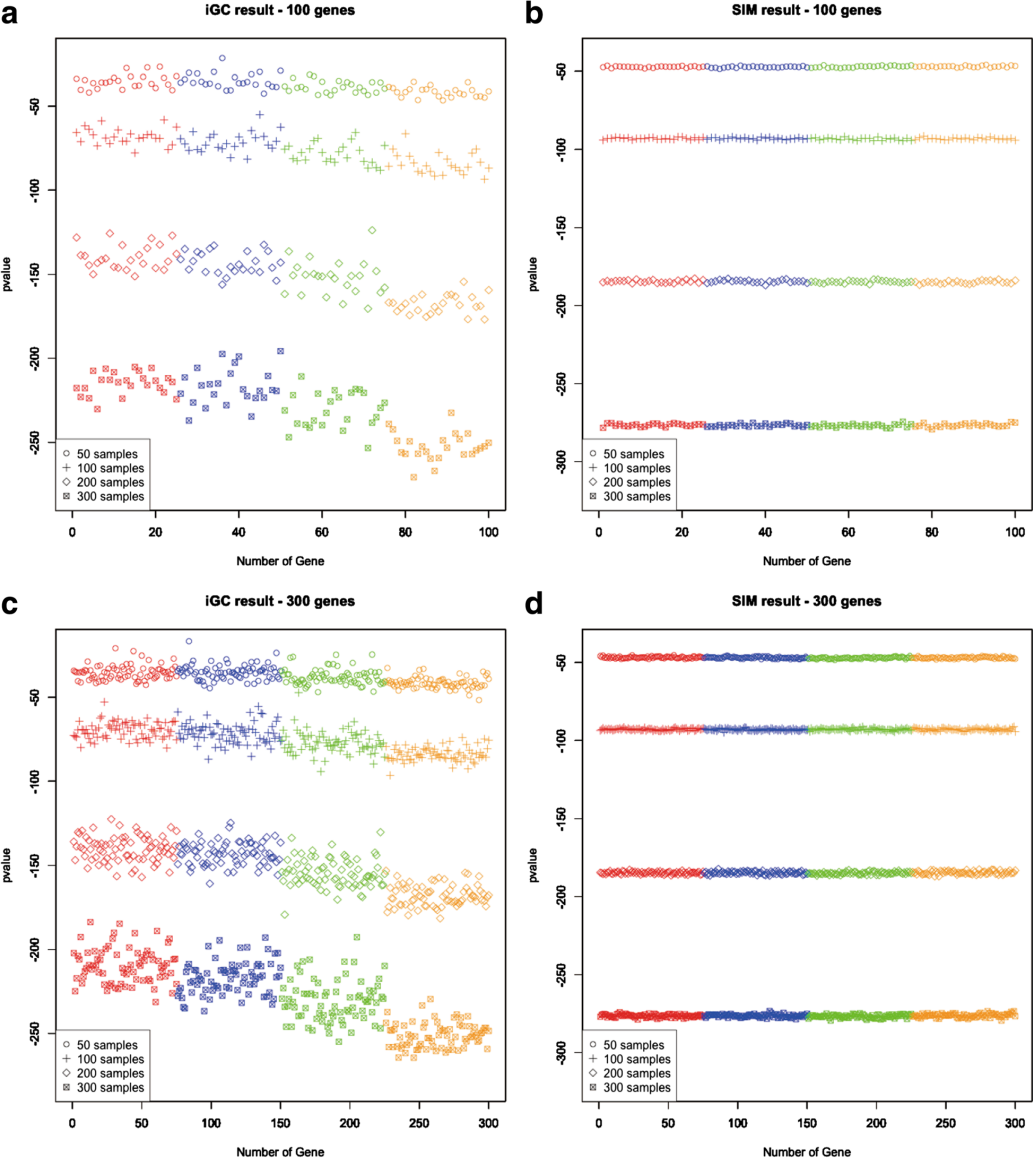
The distributions of *p*-values obtained from the iGC and SIM packages under different scenarios. Four sample sizes were simulated (*N* = 50, 100, 200 and 300) along with two numbers of genes were simulated (*N* = 100 for (**a**) and (**b**), *N* = 300 for (**c**) and (**d**)). Four groups with different Pearson correlation coefficients between CN and GE are illustrated using different colors: *red*, *r* = 0; *blue*, *r* = 0-0.3; *green*, *r* = 0.3-0.7; *orange*, *r* = 0.7-1. Each group has the same number of genes and the four groups are sorted based on the Pearson correlation coefficients

The performance statistics of the two packages are summarized in Table 1. Notably, the sensitivity values from iGC in all scenarios ranged from 0.63–0.84 and the median values were around 0.7, whereas the sensitivity values from SIM ranged from 0.18–0.36. Moreover, the specificity values from iGC were all higher than 0.86, and most of them were higher than 0.9. On the other hand, the specificity values from SIM were all less than 0.8. Therefore, the simulation data demonstrated that the iGC package is effective in identifying genes showing high correlation between their GE and CN. In addition, the *p*-values of the genes in the four groups showing different Pearson correlation coefficients are illustrated in Fig. 2. Notably, at each sample size, the *p*-values of the genes reported from the iGC package decreased as their correlation became higher (Fig. 2a and c). On the contrary, the *p*-values of the genes from SIM showed no change at higher correlation values (Fig. 2b and d). In conclusion, the simulation data demonstrated that the iGC package is able to discriminate genes showing high correlation between their CN and GE from genes showing moderate or low correlation.

**Table 1:** The performance of the iGC and SIM packages in different scenarios

Scenario	Gene number	Sample size	iGC sensitivity (mean ± sd)	iGC specificity (mean ± sd)	SIM sensitivity (mean ± sd)	SIM specificity (mean ± sd)
1	100	50	0.6293 ± 0.075	0.8764 ± 0.025	0.2582 ± 0.1118	0.7527 ± 0.0373
2	100	100	0.7283 ± 0.0651	0.9094 ± 0.0217	0.3503 ± 0.0817	0.7834 ± 0.0272
3	100	200	0.807 ± 0.0562	0.9357 ± 0.0187	0.3766 ± 0.0834	0.7922 ± 0.0278
4	100	300	0.8436 ± 0.0531	0.9479 ± 0.0177	0.3982 ± 0.0831	0.7994 ± 0.0277
5	300	50	0.6326 ± 0.0426	0.8775 ± 0.0142	0.2058 ± 0.0592	0.7353 ± 0.0197
6	300	100	0.7287 ± 0.0372	0.9096 ± 0.0124	0.2735 ± 0.0475	0.7578 ± 0.0158
7	300	200	0.8053 ± 0.0328	0.9351 ± 0.0109	0.2553 ± 0.0454	0.7518 ± 0.0151
8	300	300	0.8415 ± 0.0313	0.9472 ± 0.0104	0.2431 ± 0.0425	0.7477 ± 0.0142

### Analyses of two real microarray datasets

To demonstrate the usage of the iGC package, two publicly available microarray datasets were analyzed. The first dataset was collected from the TCGA database and included 523 breast cancer and 58 normal samples [23]. The second dataset was released from Memorial Sloan-Kettering Cancer Center and included 193 lung adenocarcinoma patients [24]. Both datasets contain paired GE and CN data from the same individual. Default parameters shown in the “Implementation” section were utilized here. Student’s *t*-test with unequal variance was used to identify differentially expressed genes (*P* < 0.001) that were significantly associated with CNA.

### Comparison of iGC and Venn diagram approaches in the TCGA dataset of breast cancer

The top three significant genes with CN gain or loss identified in the TCGA dataset are shown in Table 2. For each gene, the average GE levels of the cancerous samples in the different CNA groups (G, L, N) were calculated by subtracting the GE levels obtained from the normal samples. Obviously, the three genes showing CN gain had higher average GE values in the corresponding cancerous samples, whereas the three genes with CN loss had lower average GE values (Table 2). Among the identified genes shown in Table 2, previous studies demonstrated that SETDB1 [25, 26], GSTM1 [27, 28] and LYN [29] were located in the CNA regions in breast cancer patients. To compare the results obtained from the iGC package with that from Venn diagram, we did both analyses in the TCGA dataset.

**Table 2:** The top three significant genes with copy number gain or loss in the TCGA dataset

Genes	GE mean gain	GE mean loss	GE mean neutral	GE mean diff.	CNA prop. gain	CNA prop. loss	*t*-test	FDR^a^
GNPAT (G)	0.372	−1.048	−0.200	0.601	0.558	0.015	3.01E-61	3.43E-58
SETDB1 (G)	0.505	NA	−0.056	0.562	0.556	0	4.55E-58	2.60E-55
ANGEL2 (G)	0.588	−0.664	0.032	0.577	0.549	0.013	1.30E-55	4.93E-53
GSTM1 (L)	0.588	−0.961	0.026	−1.281	0.310	0.409	1.02E-33	5.10E-31
TOX (L)	−2.599	−3.237	−2.455	−0.777	0.023	0.337	1.64E-19	4.10E-17
LYN (L)	0.224	−0.134	0.279	−0.410	0.034	0.314	4.16E-19	6.92E-17

*GE* gene expression, *CNA* copy number alteration, *Diff* difference, *Prop* proportion, *FDR* false discovery rate, *NA* not available

^a^Genes were ordered based on the FDR values

**Fig. 3:**
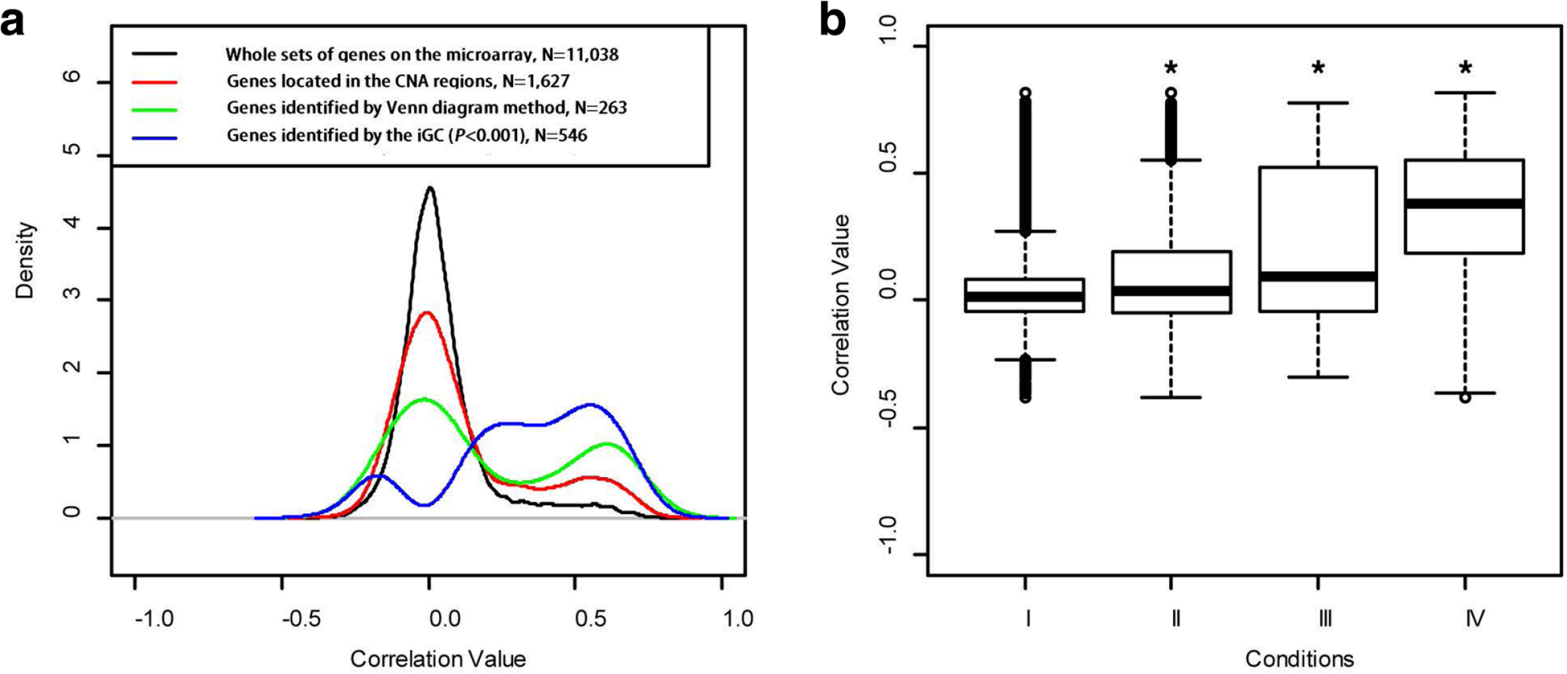
Pearson correlation coefficients between GE and CN in the TCGA breast cancer dataset in (**a**) a *Gaussian density plot* and (**b**) a *boxplot*. Four conditions were evaluated: I) the whole set of genes on the microarray, II) the subset of genes located in the CNA regions, III) the genes identified by the Venn diagram method, and IV) the genes identified by the iGC package (^*^ *P* < 0.001)

The genes showing CN gain and loss in at least 20% of the samples were analyzed further, which resulted in 2110 genes. Subsequently, Student’s *t*-test with unequal variance was performed between cancer and normal samples to identify differentially expressed genes. A total of 2070 differentially expressed genes were selected (*P* < 10^−18^). The Venn diagram approach reported 263 genes were in common among the genes with CNAs and differential expression. Alternatively, the iGC package identified 218 genes in common (*P* < 10^−18^). The two approaches simultaneously identified 78 genes, suggesting the similarity of the methods, at this stage, is 30–35%. Next, the Pearson correlation coefficients were calculated to evaluate the correlation between GE and CN in four groups of genes: the whole set of genes on the microarray, the subset of genes located in the CNA regions in >20% of the samples, the CNA-driven genes identified by iGC, and the CNA-driven genes identified by the Venn diagram approach (Fig. 3). For the whole set of genes in the TCGA sample and the subset of genes located in CNA regions, most of the correlations are between −0.2 and 0.2, suggesting their GE levels are not correlated with CNAs. Although the Venn diagram approach does have a higher proportion of genes with positive correlations, its primary peak of distribution still centers on zero. In contrast, the genes identified by the iGC approach have either positive or negative correlations, and very few genes with zero correlation are identified by the iGC approach. Genes identified by the iGC approach had significantly higher correlation values, as shown in Fig. 3b, suggesting its effectiveness to identify CNA-driven genes. However, the Venn diagram approach cannot provide the ranking of identified genes, making it difficult to select genes for advanced analyses.

**Fig. 4:**
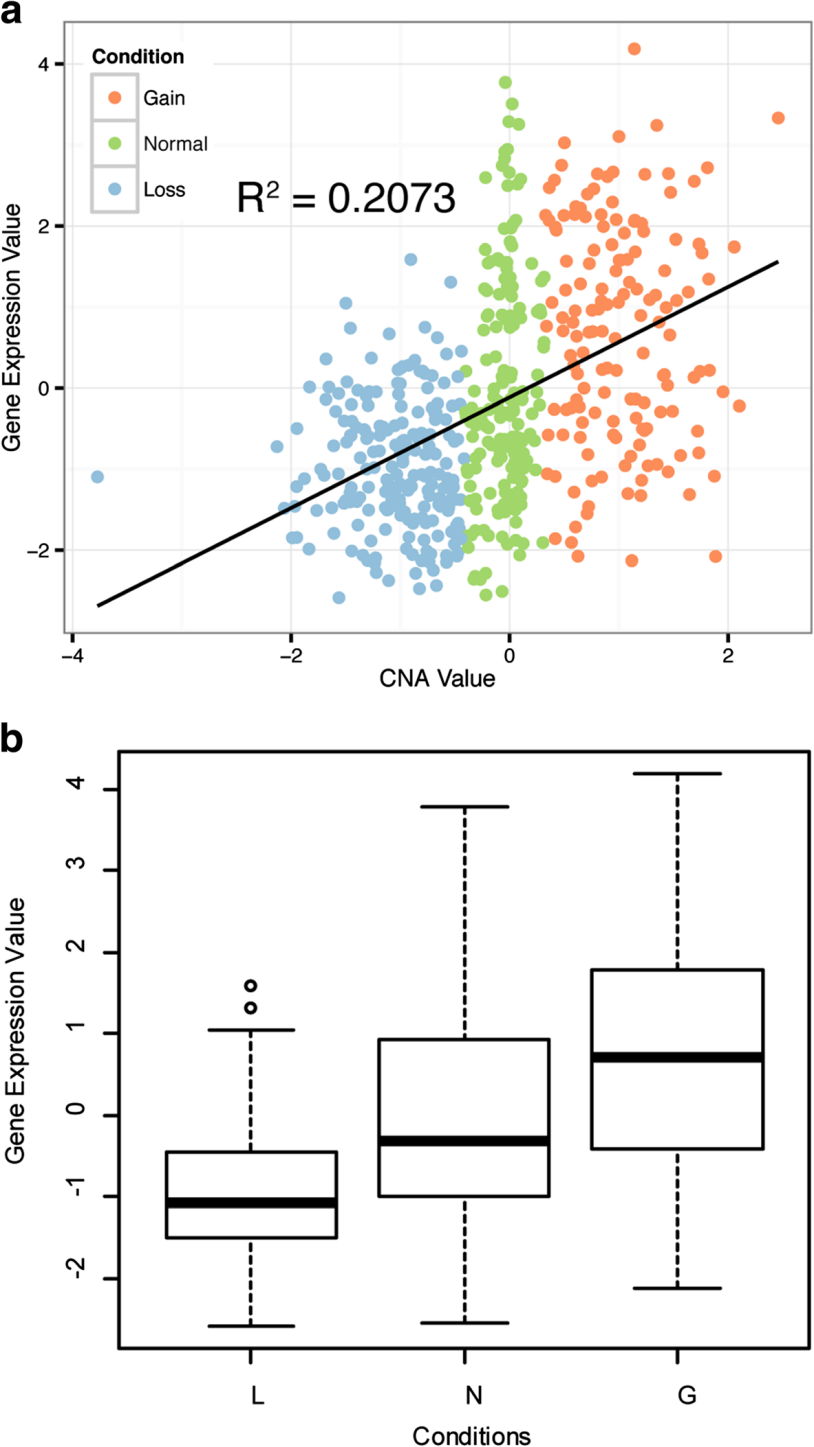
The correlation between GE and CN for the gene GSTM1 in the TCGA breast cancer dataset, presented as (**a**) a *scatter plot* and (**b**) a *boxplot*. L, CN loss; N, no gain or loss in CN; G, CN gain

To further compare the two approaches, Fisher’s exact tests were performed for each gene by classifying the 581 TCGA samples as cancerous or normal. A total of 3683 genes were identified by the Fisher’s exact test, and the iGC and Venn diagram approaches were performed on them. The iGC approach identified 546 significant genes (*P* < 0.001) whereas the Venn diagram approach reported 393 genes based on 2070 differentially expressed genes (*P* < 10^−18^). The two approaches reported 141 genes in common, indicating 25–35% similarity. However, some important genes showing correlation between GE and CN were missing from the results of the Venn diagram approach. For example, GSTM1, which showed CNAs in 70% of the samples, including 30% with CNA gains and 40% with CNA losses, was only identified by the iGC package. The paired GE and CN of GSTM1 is shown in Fig. 4. A moderate correlation between GE and CN (Pearson correlation coefficient, *r* = 0.46, *R* ^*2*^ = 0.2073, *P* = 2.2 × 10^−16^) is shown in Fig. 4a, and expression levels differed among the three groups based on CNA status (Fig. 4b).

The genes identified by the iGC package showed significant correlation between GE and CN, indicating the iGC package is able to identify differentially expressed genes driven by CNAs. It is worth mentioning that the iGC package cannot identify genes showing CNA in all samples because no appropriate control exists for performing comparisons in such a situation. Lastly, some genes showing negative correlation between GE and CN (Fig. 3b) may result from other, non-CNA-related regulatory mechanisms [30–33].

### Analysis of a microarray dataset of lung adenocarcinoma

**Fig. 5:**
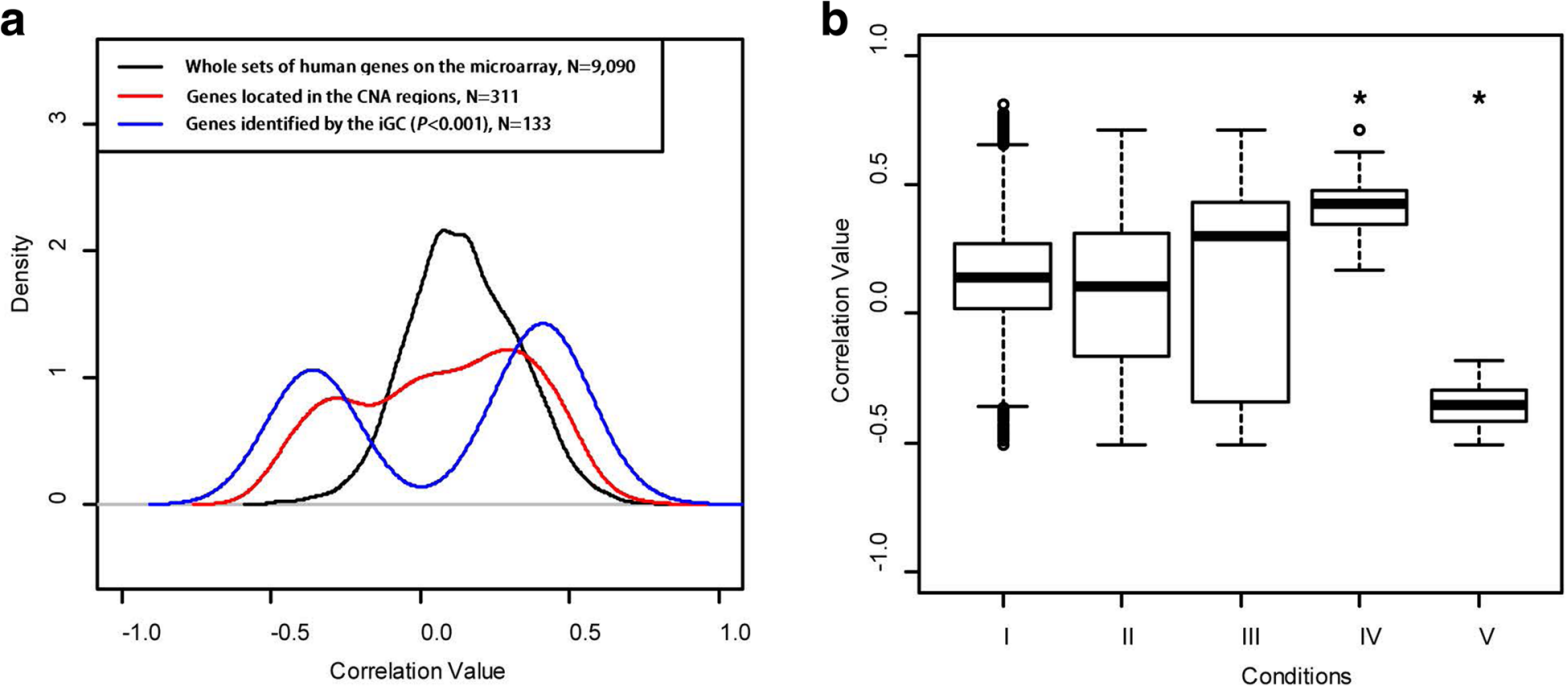
Pearson correlation coefficients between GE and CN in the lung adenocarcinoma dataset in (**a**) a *Gaussian density plot* and (**b**) a *boxplot*. Three conditions were evaluated: I) the whole set of genes on the microarray, II) the subset of genes located in the CNA regions, and III) the genes identified by the iGC package (^*^ *P* < 0.001). Conditions IV and V were split from condition III, where IV) contained genes with positive correlations between GE and CNA and V) contained genes with negative correlations

In addition to the breast cancer dataset, the iGC approach was applied to 193 lung adenocarcinoma samples with paired GE and CN microarrays, which were released from Memorial Sloan-Kettering Cancer Center [24]. Similar to the findings in the breast cancer samples, correlations between GE and CN in the whole set of human genes and in the subset of genes located in the CNA regions in the lung cancer sample were centered on zero (Fig. 5a). Although the correlations of the genes identified by the iGC approach showed no significant differences from the set of whole human genes or the subset of genes in the CNA regions (Fig. 5b), the Gaussian density plot of them illustrated that two peaks centering on 0.4 and −0.4 can be observed (Fig. 5a). That is, the genes identified by the iGC approach had either positive or negative correlation. When the iGC genes were divided into two groups based on the direction of their correlation, significant differences were observed (Fig. 5b). To focus on the purpose of integration of GE and CN, only genes with positive correlations were subjected to further analyses. The three most significant genes with CN gain or loss are shown in Table 3. Similar to the results obtained from the TCGA patients, Among them, somatic mutations in EIF1AX have been reported in cancer [34, 35]. In addition, previous studies have indicated that ALAS2 and TTTY15 are associated with cancer [36, 37].

**Table 3:** The three most significant genes with copy number gain or loss in the lung adenocarcinoma dataset

Genes	GE mean gain	GE mean loss	GE mean neutral	GE mean diff.	CNA prop. gain	CNA prop. loss	*t*-test	FDR^a^
EIF1AX (G)	8.798	9.029	8.060	0.731	0.275	0.005	4.23E-21	1.21E-18
RAP2C (G)	7.599	NA	7.093	0.505	0.285	0.000	3.33E-12	4.78E-10
ALAS2 (G)	5.765	NA	6.213	−0.448	0.347	0.000	1.64E-11	1.18E-09
RPS4Y1 (L)	NA	6.507	9.472	−2.965	0.000	0.383	6.16E-28	1.54E-26
TTTY15 (L)	5.988	4.463	4.955	−0.510	0.010	0.420	1.51E-17	1.88E-16
PRKY (L)	6.408	4.800	5.154	−0.363	0.005	0.358	7.81E-17	6.51E-16

*GE* gene expression, *CNA* copy number alteration, *Diff* difference, *Prop* proportion, *FDR* false discovery rate, *NA* not available

^a^Genes were ordered based on the FDR values

Thus, those genes that have positive correlation between GE and CNA identified by the iGC package were categorized condition IV (*n* = 78), and genes that have negative ones were categorized as condition V (*n* = 55). The genes of conditions IV and V showed significantly higher absolute correlation values (*P* < 1.94E-37 and *P* < 4.61E-47 respectively), indicating that our iGC package is capable of identifying differentially expressed genes driven by CNAs.

## Conclusions

The iGC package is capable of identifying differentially expressed genes driven by CNAs. In addition to microarray datasets, next-generation sequencing data can be analyzed in the iGC package. We believe that such approaches considering individual changes in both the genome and the transcriptome will become more popular concurrent with the advancement in high-throughput technologies.

## Availability and requirements

- **Project name:** iGC (Additional files 1, 2 and 3)
- **Project home page:** http://bioconductor.org/packages/iGC/
- **Operating system (s):** Platform independent
- **Programming language:** R
- **Other requirements:** *R* (> = 3.2.0), Bioconductor (> = 3.2), plyr, data.table
- **License:** GNU GPLv2
- **Any restrictions to use by non-academics:** None
- The two microarray datasets [17, 24] analyzed in this study are in the public domain and the raw files can be retrieved from their original websites.

## Additional files

Additional file 1: The source codes and example data of the package iGC in R. (GZ 2818 kb)
Additional file 2: The tutorial of the package iGC. (PDF 129 kb)
Additional file 3: The introduction page of the package iGC. (HTML 96 kb)

## Abbreviations

CNA: Copy number alteration
GE: Gene expression
GEO: Gene Expression Omnibus
iGC: Integrated analysis of GE and CNA
TCGA: The Cancer Genome Atlas
