# Supplementary figures and images for: iGC—an integrated analysis package of gene expression and copy number alteration
Source: BMC Bioinformatics. 2017 Jan 14;18:35. doi: 10.1186/s12859-016-1438-2 (PMC5237550; doi:10.1186/s12859-016-1438-2)

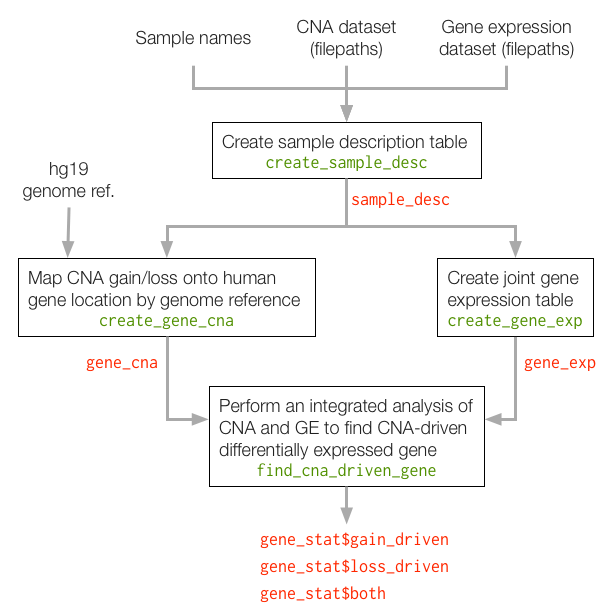

Supplement: Additional file 1: — The source codes and example data of the package iGC in R. (GZ 2818 kb) [file 12859_2016_1438_MOESM1_ESM.gz › iGC/vignettes/pics/analysis_workflow.png]
