# Supplementary material for: iGC—an integrated analysis package of gene expression and copy number alteration
Source: BMC Bioinformatics. 2017 Jan 14;18:35. doi: 10.1186/s12859-016-1438-2 (PMC5237550; doi:10.1186/s12859-016-1438-2)
Supplement: Additional file 2: — The tutorial of the package iGC. (PDF 129 kb) [file 12859_2016_1438_MOESM2_ESM.pdf]

Package 'iGC'

August 19, 2015

Type Package

Title An integrated analysis package of Gene expression and Copy number alteration

Version 0.99.2

Description This package is intended to identify differentially expressed genes driven by Copy Number Alterations from samples with both gene expression and CNA data.

biocViews Software, Biological Question, DifferentialExpression, GenomicVariation, AssayDomain, CopyNumberVariation, GeneExpression, ResearchField, Genetics, Technology, Microarray, Sequencing, WorkflowStep, MultipleComparison

License GPL-2

URL http://github.com/ccwang002/iGC

BugReports http://github.com/ccwang002/iGC/issues

VignetteBuilder knitr

Enhances doMC

Suggests BiocStyle, knitr, rmarkdown

Imports plyr, data.table

Depends R (>= 3.2.0)

LazyData true

NeedsCompilation no

Author Yi-Pin Lai [aut],
Liang-Bo Wang [aut, cre],
Tzu-Pin Lu [aut],
Eric Y. Chuang [aut]

Maintainer Liang-Bo Wang <r02945054@ntu.edu.tw>

R topics documented:

create_gene_cna 2
create_gene_exp 4
create_sample_desc 5
direct_gene_cna 7
find_cna_driven_gene 8
hg19DBNM 10
iGC 11

Index **12**

`create_gene_cna` *Load and map CNA gain/loss onto human gene location by genome reference*

Description

The function reads through in all sample CNA data given by the sample description `sample_desc` and returns a joint CNA gain/loss table based on gene regions across samples.

Usage

```
create_gene_cna(sample_desc, gain_threshold = log2(2.5) - 1,
  loss_threshold = log2(1.5) - 1, read_fun = NULL, progress = TRUE,
  progress_width = 48, parallel = FALSE, ...)
```

Arguments

`sample_desc`	data.table object created by create_sample_desc.
`gain_threshold`	CNA expression above this will be considered as gain region. By default $\log_2 2.5 - 1$
`loss_threshold`	CNA expression below this will be considered as loss region. By default $\log_2 1.5 - 1$
`read_fun`	Custom reader function, see its own section for more detail.
`progress`	Whether to display a progress bar. By default `TRUE`.
`progress_width`	The text width of the shown progress bar. By default is 48 chars wide.
`parallel`	Enable parallelism by plyr. One has to specify a parallel engine beforehand. See example for more information.
`...`	Arguments passed to the custom reader function specified in `read_fun`.

Details

A gene is considered to have CNA gain if the overlapped CNA record expression is higher than the given threshold. Similarly, a gene is considered CNA loss if the overlapped CNA record is lower than the given threshold. If multiple CNA records map onto the same gene region with both gain and loss, the majority wins. If none of the records map to the gene, NA is given.

By default it assumes the data to be of TCGA level 3 file format. For other data formats (e.g. raw data or other experiments from GEO), one should implement a custom reader function that accepts the filepath as the first argument. See section *Custom reader function* for full specification.

Currently the package ships a custom genome reference hg19, hg19DBNM, for gene region look up. Each gene's region is defined by the widest splicing form it has in NCBI curated records. The defined region includes intron regions. This limitation may change in the future.

Value

data.table of CNA gain/loss on each gene region for all samples, whose rows represent regions of genes and columns represent sample names. First column `GENE` contains the corresponding gene names.

Custom reader function

Custom reader function is given by `read_fun = your_reader_fun`. It takes the filepath to CNA data as the first argument and returns a data.table with at least the following four columns: `Chromosome`, `Start`, `End`, and `Segment_Mean` of type character, integer, integer and numeric respectively.

Rest arguments of `create_gene_cna(...)` will be passed to this reader function.

Note: all string-like columns should **NOT** be of type `factor`. Remember to set `stringsAsFactors = FALSE`.

See Also

`read.table` and `fread` for custom reader function implementation; `create_sample_desc` for creating sample description. If the gene information already exists in the data, try direct_gene_cna to skip the genome reference lookup.

Examples

```
## Use first three samples of the builtin dataset

sample_root <- system.file("extdata", package = "iGC")
sample_desc_pth <- file.path(sample_root, "sample_desc.csv")
sample_desc <- create_sample_desc(
    sample_desc_pth, sample_root=sample_root
)[1:3]


## Define custom reader function for TCGA level 3 gene exp. data

my_cna_reader <- function(cna_filepath) {
    cna <- data.table::fread(cna_filepath, sep = '\t', header = TRUE)
    data.table::setnames(
        cna,
        c("Sample", "Chromosome", "Start", "End", "Num_Probes", "Segment_Mean")
    )
    # pick only the needed columns
    cna[, .(Chromosome, Start, End, Segment_Mean)]
}


## Read all samples' CNA data and combined as a single table

gene_cna <- create_gene_cna(
    sample_desc,
    gain_threshold = log2(2.3) - 1, loss_threshold = log2(1.7) - 1,
    read_fun = my_cna_reader,
)
gene_cna[GENE %in% c("BRCA2", "TP53", "SEMA5A"), ]


## Not run:
## To boost the speed, utilize parallelization

doMC::registerDoMC(4)  # number of CPU cores
gene_cna <- create_gene_cna(
    sample_desc,
    gain_threshold = log2(2.3) - 1, loss_threshold = log2(1.7) - 1,
```

```
    read_fun = my_cna_reader,
    parallel = TRUE
)

## End(Not run)
```

`create_gene_exp` *Create an joint gene expression table of all samples*

Description

The function reads in all gene expression data given by the sample description `sample_desc` and return a joint expression table of all samples.

Usage

```
create_gene_exp(sample_desc, read_fun = NULL, progress = TRUE,
  progress_width = 48, ...)
```

Arguments

`sample_desc`	data.table object created by create_sample_desc.
`read_fun`	Custom reader function, see its own section for more detail.
`progress`	Whether to display a progress bar. By default `TRUE`.
`progress_width`	The text width of the shown progress bar. By default is 48 chars wide.
`...`	Arguments passed to the custom reader function specified in `read_fun`.

Details

By default it assumes the data to be of TCGA level 3 file format. However, nearly all real world data fail to have the same format as TCGA. In this case, one needs to tell the function how to parse the data by implementing a custom reader function that accepts the filepath as the first argument. See Detail section for full specification. The function naively concatenates all return expression *as if all gene expressions are stated in the same gene order* as columns in a new data.table.

Value

data.table of all samples gene expression, whose rows are gene expression and columns are sample names. First column `GENE` contains the corresponding gene names.

Custom reader function

Custom reader function is given by `read_fun = your_reader_fun`. It takes the filepath as the first argument and return a data.table with the first two columns being `GENE` and `Expression` of type character and double.

The output joint gene expression table has first column `GENE` store the gene name, which are are determined by the first sample being evaluated.

Rest arguments of `create_gene_exp(...)` will be passed to this reader function.

Note: all string-like columns should **NOT** be of type `factor`. Remember to set `stringsAsFactors = FALSE`.

Note

The function assumes row order for all samples' gene expressions are the same.

See Also

read.table and fread for custom reader function implementation; create_sample_desc for creating sample description.

Examples

```
## Use first three samples of the builtin dataset

sample_root <- system.file("extdata", package = "iGC")
sample_desc_pth <- file.path(sample_root, "sample_desc.csv")
sample_desc <- create_sample_desc(
    sample_desc_pth, sample_root=sample_root
)[1:3]

## Define custom reader function for TCGA level 3 data
my_gene_exp_reader <- function(ge_filepath) {
    gene_exp <- read.table(
        ge_filepath,
        header = FALSE, skip = 2,
        na.strings = "null",
        colClasses = c("character", "double")
    )
    dt <- data.table::as.data.table(gene_exp)
    data.table::setnames(dt, c("GENE", "Expression"))
}
gene_exp <- create_gene_exp(
    sample_desc,
    read_fun = my_gene_exp_reader,
    progress_width = 60
)
gene_exp[1:5]
```

---

create_sample_desc — *Create sample description table containing all required inputs*

Description

Each sample will have a unique name along with a pair of CNA and gene expression file. This function generates a table of sample descriptions by either reading an external CSV file or specifying them through separate arugments in same order.

Usage

```
create_sample_desc(sample_desc_filepath = NULL, sample_names = NULL,
  cna_filepaths = NULL, ge_filepaths = NULL, sample_root = NULL)
```

Arguments

`sample_desc_filepath`
: external sample description CSV file having at least these three columns: `Sample`, `CNA_filepath`, and `GE_filepath`. Note that the column names must be given *as is*.

`sample_names`
: character vector of distinct sample names. Samples will be referenced by the given name through out the analysis process. They should be valid R data.table column names.

`cna_filepaths`
: character vector of filepaths to CNA data.

`ge_filepaths`
: character vector of filepaths to gene expression data.

`sample_root`
: path to the root of sample data. If given, this path will be appended before all given filepaths.

Value

data.table of sample description having the following columns in order: `Sample`, `CNA_filepath`, and `GE_filepath`. Each row contains a sample's unique name and the corresponding filepaths to CNA and gene expression data.

Note

One could convert the relative file paths into absolute paths by passing the root folder path to `sample_root`.

If for some special reasons, for example gene expression of all samples have been collected or the CNA records for each gene exist, but do not have the file paths to either CNA or gene expression data, pass it with empty character vector of correct length, such as `rep('', num_samples)`.

Examples

```
## Custom sample description by specifying separate arguments

sample_names <- letters[1:5]
sample_desc <- create_sample_desc(
    sample_names = sample_names,
    cna_filepaths = file.path('cna', paste0(sample_names, '.csv')),
    ge_filepaths = file.path('ge', paste0(sample_names, '.txt'))
)
sample_desc


## Prepend the file path with a root directory /path/to/sample

create_sample_desc(
    sample_names = sample_desc$Sample,
    cna_filepaths = sample_desc$CNA_filepath,
    ge_filepaths = sample_desc$GE_filepath,
    sample_root = '/path/to/sample'
)


## Create by reading a sample description CSV file

sample_desc_pth <- system.file("extdata", "sample_desc.csv", package = "iGC")
```

```
sample_desc <- create_sample_desc(sample_desc_pth)


## Not run:
## Read a external description and append the given file paths
create_sample_desc('/path/to/desc.csv', sample_root='/path/to/sample/root')

## End(Not run)
```

`direct_gene_cna` *Load the existed CNA gain/loss based on gene location.*

Description

This function aims to complement `create_gene_cna`. Instead of mapping CNA records onto genes by genome reference, it reads the existed column containing the gene each CNA lies on. Two functions share the same interface but they have different requirement for the `read_fun` implementation.

Usage

```
direct_gene_cna(sample_desc, gain_threshold = log2(2.5) - 1,
  loss_threshold = log2(1.5) - 1, read_fun = NULL, progress = TRUE,
  progress_width = 48, parallel = FALSE, ...)
```

Arguments

`sample_desc`	data.table object created by create_sample_desc.
`gain_threshold`	CNA expression above this will be considered as gain region. By default $\log_2 2.5 - 1$
`loss_threshold`	CNA expression below this will be considered as loss region. By default $\log_2 1.5 - 1$
`read_fun`	Custom reader function, see its own section for more detail.
`progress`	Whether to display a progress bar. By default `TRUE`.
`progress_width`	The text width of the shown progress bar. By default is 48 chars wide.
`parallel`	Enable parallelism by plyr. One has to specify a parallel engine beforehand. See example for more information.
`...`	Arguments passed to the custom reader function specified in `read_fun`.

Value

data.table of CNA gain/loss on each gene region for all samples, whose rows represent regions of genes and columns are sample names. First column `GENE` contains the corresponding gene names.

Custom reader function

Similar to that of create_gene_cna, the reader function takes the filepath as the first argument. It will return a data.table with at least two columns: `GENE` and `Segment_Mean` of type `character` and `numeric` respectively.

See Also

create_gene_cna

Examples

```
require(data.table)

## Create a CNA dataset that has been already mapped onto gene regions

cna_geo_list = list(
    sample_A = data.table(
        GENE = c("TP53", "BRCA2"),
        Segment_Mean = c(1.05, -2.03)
    ),
    sample_B = data.table(
        GENE = c("TP53", "BRCA2", "NDPH1"),
        Segment_Mean = c(0.38, -1.71, 2.6)
    )
)
sample_desc <- data.table(
    Sample = paste("sample", c("A", "B"), sep = "_")
)
sample_desc$CNA_filepath <- sample_desc$Sample


## Example code for reading

read_cna_geo <- function(pth) {
    # For demonstration, file reading silently redirects
    # to list lookup
    cna_geo_list[[pth]]
}
gene_cna <- direct_gene_cna(
    sample_desc,
    read_fun = read_cna_geo, progress = FALSE
)
gene_cna
```

---

find_cna_driven_gene *Perform an integrated analysis of gene expression (GE) and copy number alteration (CNA)*

Description

The function finds CNA-driven differentially expressed gene and returns the corresponding p-value, false discovery rate, and associated statistics. The result includes three tables which collects information for gain-, loss-, and both-driven genes.

Usage

```
find_cna_driven_gene(gene_cna, gene_exp, gain_prop = 0.2, loss_prop = 0.2,
  progress = TRUE, progress_width = 32, parallel = FALSE)
```

Arguments

gene_cna	Joint CNA table from create_gene_cna.
gene_exp	Joint gene expression table from create_gene_exp.
gain_prop	Minimum proportion of the gain samples to be consider CNA-gain. Default is 0.2.
loss_prop	Minimum proportion of the loss samples to be consider CNA-loss. Default is 0.2.
progress	Whether to display a progress bar. By default TRUE.
progress_width	The text width of the shown progress bar. By default is 48 chars wide.
parallel	Enable parallelism by plyr. One has to specify a parallel engine beforehand. See example for more information.

Details

The gene is considered CNA-gain if the proportion of the sample exhibiting gain exceeds the threshold gain_prop, that is, number of samples having gain_loss = 1. Reversely, the gene is considered CNA-loss if %samples that gain_loss = -1 is below a given threshold loss_prop.

When performing the t-test, sample grouping depends on the analysis scenario being either CNA-gain or CNA-loss driven. In CNA-gain driven scenario, two groups, CNA-gain and the other samples, are made. In CNA-loss driven scenario, group CNA-loss and the others are made. Genes that appear in both scenarios will be collected into a third table and excluded from their original tables.

See the vignette for usage of this function by a thorough example.

Value

List of three data.table objects for CNA-driven scenarios: gain, loss, and both, which can be accessed by names: ‘gain_driven‘, ‘loss_driven‘ and ‘both‘.

Examples

```
require(data.table)

## Create gene_exp and gene_cna manually. The following shows an example
## consisting of 3 genes (BRCA2, TP53, and GNPAT) and 5 samples (A to E).

gene_exp <- data.table(
    GENE = c("BRCA2", "TP53", "GNPAT"),
    A = c(-0.95, 0.89, 0.21), B = c(1.72, -0.05, NA),
    C = c(-1.18, 1.15, 2.47), D = c(-1.24, -0.07, 1.2),
    E = c(1.01, 0.93, 1.54)
)
gene_cna <- data.table(
    GENE = c("BRCA2", "TP53", "GNPAT"),
    A = c(1, 1, NA), B = c(-1, -1, 1),
    C = c(1, -1, 1), D = c(1, -1, -1),
    E = c(0, 0, -1)
)


## Find CNA-driven genes

cna_driven_genes <- find_cna_driven_gene(
```

```
    gene_cna, gene_exp, progress=FALSE
)

# Gain driven genes
cna_driven_genes$gain_driven

# Loss driven genes
cna_driven_genes$loss_driven

# Gene shown in both gain and loss records
cna_driven_genes$both
```

---

hg19DBNM *hg19-RefSeq*

Description

The human genome reference used here is RefSeq transcripts in version hg19 from UCSC Genome Browser. The transcripts with NM marker ID, which are protein-codeing, were selected to be our reference database and provided as hg19DBNM.rda.

Usage

```
hg19DBNM
```

Format

A data frame with 39997 rows and 7 variables:

Marker.ID RefSeq name with its corrsponding gene symbol

Chromosome 1-22, X and Y

Start starting position, in basepair number

Stop ending position, in basepair number

Strand positive or negative strand, in + or - symbols

Gene.Symbol Gene name

Transcript RefSeq name

Details

This reference provides region information, including chromosome number, starting position, ending position, strand and gene symbols, for converting copy number alteration data into human genes.

Value

data.table

Source

UCSC Genome Browser: http://hgdownload.cse.ucsc.edu/downloads.html

iGC *iGC: an integrated analysis package of gene expression and copy number alteration*

Description

The iGC package is used to identify CNA-driven differentially expressed genes. The iGC package provides four categories of important functions: ‘create_sample_desc‘, ‘create_gene_ex‘, ‘create_gene_cna‘ and ‘find_cna_drive_gene‘.

create_sample_desc

The create_sample_desc function is provided for creating a sample description table containing all required inputs.

create_gene_exp function

The create_gene_exp function is used to rearrange the input gene expression files into a gene expression list of entire samples.

create_gene_cna function

The create_gene_cna function maps CNA data to human genes and then defines the mapped human genes as CN gain or loss based on the CN threshold, whose default values are set as 2.5 for gain and 1.5 for loss. These mapped genes will be assigned values in +1, -1 or 0, where +1 stands for CNA-gain, -1 stands for CNA-loss and 0 stands for neutral.

find_cna_driven_gene function

The find_cna_driven_gene function identifies CNA-driven differentially expressed genes. The input mapped genes remain for further analyses if its ratio of the number of CN changed samples, CNA-gain (G) or CNA-loss (L), to the number of total samples is larger than a given threshold. Here the default setting is that only genes showing CNAs in at least 20 statistical tests, T-test and Wilcoxon rank sum test, are performed in the GE level by classifying the samples as G and L plus Nertral (N) groups or L and G plus N groups, depending on the CN of the interested gene increases or decreases.

Index

∗Topic **datasets**
  hg19DBNM, 10

create_gene_cna, 2, *7–9*
create_gene_exp, 4, *9*
create_sample_desc, *2–5*, 5, *7*

data.table, *2*, *7*
direct_gene_cna, *3*, 7

find_cna_driven_gene, 8
fread, *3*, *5*

hg19DBNM, *2*, 10

iGC, 11
iGC-package *(*iGC*)*, 11

read.table, *3*, *5*
