# Supplementary material for: iGC—an integrated analysis package of gene expression and copy number alteration
Source: BMC Bioinformatics. 2017 Jan 14;18:35. doi: 10.1186/s12859-016-1438-2 (PMC5237550; doi:10.1186/s12859-016-1438-2)
Supplement: Additional file 3: — The introduction page of the package iGC. (HTML 96 kb) [file 12859_2016_1438_MOESM3_ESM.html]

Introduction to iGC


# Introduction to iGC

#### *iGC Developers*

#### *2015-08-16*

- Installation
- General Workflow
- Data Source
  - Gene expression
  - CNA
  - Custom reader function
- Usage Example
  - Example Data Source
  - Sample Description Generation
  - Joint Gene Expression Table
  - Joint CNA Status Table Mapped onto Gene Locations
  - CNA-driven Differentially Expressed Genes Identification
- Q and As

This document guides one through all available functions of the `iGC` package. Package iGC aims to analyze gene expression (GE) and copy number alteration (CNA) (iGC) concurrently.

Traditional CNA analysis method is to investigate different types of samples and integrate their results by Venn diagrams. Challenges arise, however, when the low reproducibility and inconsistency are observed across multiple platforms. To address these issues, iGC tests gene expression profiles and copy number variation simultaneously.

For more information about the method iGC uses, please refer to our publication: Yi-Pin Lai, Liang-Bo Wang, Liang-Chuan Lai, Mong-Hsun Tsai, Tzu-Pin Lu, Eric Y Chuang. iGC–an integrated analysis package of Gene expression and Copy number alteration, *Bioinfomatics* (publication pending).

## Installation

iGC is on Bioconductor and can be installed following standard installation procedure.

```
source("http://www.bioconductor.org/biocLite.R")
biocLite("iGC")
```

To use,

```
library(iGC)
```

## General Workflow

The general workflow can be summarized as follows,

Basically there are four steps, corresponding to four R functions, to complete the analysis:

1. Specify sample and their data relations
2. Read and organize gene expression files
3. Read and organize CNA files based on gene regions
4. Identify CNA-driven differentially expressed genes

## Data Source

As shown in the workflow, samples of *paired* CNA and gene expression data are required for the analysis. Since iGC reads CNA and gene expression data at gene level or chromosome location, data sources are platform and technology agnostic, that is, data from either microarrary or next-generation sequencing are acceptable. However, it also implies some standard preprocessing steps are required to convert the raw readings into interpretable data.

### Gene expression

For each sample, the data should have at least two columns, gene and expression:

```
GENE  Expression
A     0.1
B     -0.5
C     0.4
```

iGC reads all the samples’ gene expression by `create_gene_exp` and make a joint gene expression table,

```
GENE  SampleA  SampleB ...
A         0.1     0.5
B        -0.5     2.1
C         0.4      NA
```

That means, if such joint expression table exists, one could skip this step.

### CNA

iGC accepts two kinds of the CNA data format, chromsome location based and gene based, which are handled by `create_gene_cna` and `direct_gene_cna` respectively. For chromosome location based CNA records, each sample’s data should has at least the following columns,

```
Choromsome  Start  End    Expression
1           1000   5030          2.5
10          10     2560         -1.3
X           12345  14200         3.3
```

iGC will convert this format of records into gene based format by looking up the genome reference, which currently supports hg19 only (see Q&A for more info and future developemnt plan).

For gene based CNA records, data format resembles the gene expression data,

```
GENE   Expression
A             2.5
B             2.5
C            -1.3
```

In the end, iGC reads all samples’ CNA records and make a joint CNA table. Different from the gene expression processing, CNA records are additionally converted to CNA gain/loss events by given thresholds.

```
GENE  SampleA  SampleB  ...
A           0        0
B          -1       -1
C           0        1
```

If such joint table already existed one could skip this step as well.

### Custom reader function

To support various kinds of data formats, all `create_gene_exp`, `create_gene_cna`, and `direct_gene_cna` accept an arugment `read_fun`, a function to read sample data, to customize how the data should be read. Please refer to their documentation to find out the implementation details.

## Usage Example

Here we show an example using the data shipped together with iGC.

### Example Data Source

To demonstrate the usage of the `iGC` package, the package comes with 50 breast cancer samples, which are selected from a microarray dataset of total 523 breast cancer samples from TCGA level 3.

For each sample, it contains a paired gene expression (GE) and the copy number (CN) data. The GE data was conducted by Agilent G4502A platform and the CN data was generated from Genome Wide SNP platform.

### Sample Description Generation

First, a sample description table is created to connect sample names with their data. It can be stored as a CSV file with three columns: `Sample`, `CNA_filepath`, and `GE_filepath`.

```
sample_desc_pth <- system.file("extdata", "sample_desc.csv", package = "iGC")
sample_desc <- create_sample_desc(sample_desc_pth)
```

Alternatively, one can pass three separate character vectors to create the same table,

```
sample_desc <- create_sample_desc(
  sample_names = sample_desc$Sample,
  cna_filepaths = sample_desc$CNA_filepath,
  ge_filepaths = sample_desc$GE_filepath
)
```

```
head(sample_desc)
```

```
##              Sample
## 1: TCGA-AO-A0JL-01A
## 2: TCGA-BH-A0HF-01A
## 3: TCGA-BH-A0HK-01A
## 4: TCGA-AO-A0JF-01A
## 5: TCGA-BH-A0DP-01A
## 6: TCGA-AO-A0JJ-01A
##                                                                                                                 CNA_filepath
## 1: /private/var/folders/by/bp5trzl90kz4wlkyfgnn62fr0000gn/T/RtmpTmRWqB/Rinst2531770c3802/iGC/extdata/CNA/A04_697150.hg19.txt
## 2: /private/var/folders/by/bp5trzl90kz4wlkyfgnn62fr0000gn/T/RtmpTmRWqB/Rinst2531770c3802/iGC/extdata/CNA/A05_697032.hg19.txt
## 3: /private/var/folders/by/bp5trzl90kz4wlkyfgnn62fr0000gn/T/RtmpTmRWqB/Rinst2531770c3802/iGC/extdata/CNA/A07_697200.hg19.txt
## 4: /private/var/folders/by/bp5trzl90kz4wlkyfgnn62fr0000gn/T/RtmpTmRWqB/Rinst2531770c3802/iGC/extdata/CNA/A09_697146.hg19.txt
## 5: /private/var/folders/by/bp5trzl90kz4wlkyfgnn62fr0000gn/T/RtmpTmRWqB/Rinst2531770c3802/iGC/extdata/CNA/B01_697160.hg19.txt
## 6: /private/var/folders/by/bp5trzl90kz4wlkyfgnn62fr0000gn/T/RtmpTmRWqB/Rinst2531770c3802/iGC/extdata/CNA/B03_697186.hg19.txt
##                                                                                                                                         GE_filepath
## 1: /private/var/folders/by/bp5trzl90kz4wlkyfgnn62fr0000gn/T/RtmpTmRWqB/Rinst2531770c3802/iGC/extdata/GE/US82800149_251976012210_S01.tcga_level3.txt
## 2: /private/var/folders/by/bp5trzl90kz4wlkyfgnn62fr0000gn/T/RtmpTmRWqB/Rinst2531770c3802/iGC/extdata/GE/US82800149_251976012504_S01.tcga_level3.txt
## 3: /private/var/folders/by/bp5trzl90kz4wlkyfgnn62fr0000gn/T/RtmpTmRWqB/Rinst2531770c3802/iGC/extdata/GE/US82800149_251976012510_S01.tcga_level3.txt
## 4: /private/var/folders/by/bp5trzl90kz4wlkyfgnn62fr0000gn/T/RtmpTmRWqB/Rinst2531770c3802/iGC/extdata/GE/US82800149_251976012517_S01.tcga_level3.txt
## 5: /private/var/folders/by/bp5trzl90kz4wlkyfgnn62fr0000gn/T/RtmpTmRWqB/Rinst2531770c3802/iGC/extdata/GE/US82800149_251976012261_S01.tcga_level3.txt
## 6: /private/var/folders/by/bp5trzl90kz4wlkyfgnn62fr0000gn/T/RtmpTmRWqB/Rinst2531770c3802/iGC/extdata/GE/US82800149_251976012204_S01.tcga_level3.txt
```

### Joint Gene Expression Table

Second, we join all samples’ gene expression as one table.

```
gene_exp <- create_gene_exp(sample_desc, progress = FALSE)
```

`create_gene_exp` comes with a builtin reader function to read in the gene expression data. However for formats that it fails to recognize, one can define one’s own reader function through `read_fun`,

```
gene_exp <- create_gene_exp(
  sample_desc,
  read_fun = read.table,
  progress = TRUE, progress_width = 60,
  # arugments passed to the customized read_fun (here is read.table)
  header = FALSE,
  skip = 2,
  na.strings = "null",
  colClasses = c("character", "double")
)
```

Note that arguments `header`, `skip`, `na.srings`, and `colClasses` are not used by `create_gene_exp` but passed to `read.table`, the custom reader function defined here, directly.

We select the expression of gene TP53, BRCA1, and NFKB1 for the first 9 samples (first column contains gene names).

```
gene_exp[GENE %in% c('TP53', 'BRCA1', 'NFKB1'), 1:10, with=FALSE]
```

```
##     GENE TCGA-AO-A0JL-01A TCGA-BH-A0HF-01A TCGA-BH-A0HK-01A
## 1:  TP53       -0.9326667       -0.4386667       -0.5056667
## 2: BRCA1       -2.0526667       -2.0460833       -2.0060833
## 3: NFKB1        1.1812000        1.3117000        0.8047000
##    TCGA-AO-A0JF-01A TCGA-BH-A0DP-01A TCGA-AO-A0JJ-01A TCGA-A8-A0A6-01A
## 1:       -0.1083333        -0.182500       -0.3288333        -0.535500
## 2:       -2.0717500        -1.448833       -0.9750000        -1.673083
## 3:        1.1610000         1.490200        1.5671000         1.358000
##    TCGA-AO-A0JM-01A TCGA-BH-A0HB-01A
## 1:      -0.06183333       -0.7593333
## 2:      -1.78058333       -2.3987500
## 3:       0.62590000        0.8151000
```

### Joint CNA Status Table Mapped onto Gene Locations

Thirdly, the CNA data is read, collected, and mapped on to human gene locations using genome reference hg19. Each gene will be evaluated as CNA-gain (1), CNA-loss (-1), and neutral (0). Threshold can be set to tune the level of CNA determined as gain or loss.

Here we set the threshold of 2.4 for gain and 1.6 for loss events.

```
my_cna_reader <- function(cna_filepath) {
  cna <- data.table::fread(cna_filepath, sep = '\t', header = TRUE)
  cna[, .(Chromosome, Start, End, Segment_Mean)]
}

gain_loss = log2(c(2.4, 1.6)) - 1
gene_cna <- create_gene_cna(
  sample_desc,
  gain_threshold = gain_loss[1], loss_threshold = gain_loss[2],
  read_fun = my_cna_reader,
  progress = FALSE
)
gene_cna[GENE %in% c('TP53', 'BRCA1', 'NFKB1'), 1:10, with=FALSE]
```

```
##     GENE TCGA-AO-A0JL-01A TCGA-BH-A0HF-01A TCGA-BH-A0HK-01A
## 1:  TP53                0                0               -1
## 2: BRCA1               -1               -1                0
## 3: NFKB1                0                1                0
##    TCGA-AO-A0JF-01A TCGA-BH-A0DP-01A TCGA-AO-A0JJ-01A TCGA-A8-A0A6-01A
## 1:                0               -1                0                0
## 2:                0                0                0                0
## 3:                0                0                0                0
##    TCGA-AO-A0JM-01A TCGA-BH-A0HB-01A
## 1:               -1               -1
## 2:               -1               -1
## 3:                0                0
```

#### Parallelization

For performance issues, one can enable parallelization to boost the process.

```
# Change 4 to match one's total CPU cores
doMC::registerDoMC(cores = 4)
gene_cna <- faster_gene_cna(
  sample_desc, gain_loss[[1]], gain_loss[[2]], parallel = TRUE
)
```

#### Read gene information directly from data

If one’s CNA data already contains gene information, try `direct_gene_cna`.

### CNA-driven Differentially Expressed Genes Identification

Lastly, run `find_cna_driven_gene` to identify differentially expressed genes driven by CNAs from samples with both proprocessed GE `gene_exp` and CNA data `gene_cna` that were obtained from the previous steps.

A threshold for proportion of the copy number changed samples (gain or loss) is given to select CNA-driven genes.

```
cna_driven_genes <- find_cna_driven_gene(
  gene_cna, gene_exp,
  gain_prop = 0.15, loss_prop = 0.15,
  progress = FALSE, parallel = FALSE
)
head(cna_driven_genes$gain_driven)
```

```
##        GENE      p_value         fdr gain_sample_prop normal_sample_prop
## 1:    TRPT1 5.515568e-07 0.002097571           0.2500             0.7500
## 2: SLC9A3R1 1.995817e-06 0.002530031           0.2500             0.6875
## 3:   SNAPC1 1.555874e-06 0.002530031           0.1875             0.6875
## 4:     PMVK 4.758945e-06 0.004524567           0.7500             0.2500
## 5:    SIRPG 6.048564e-06 0.004600538           0.1875             0.6875
## 6: CDC42EP2 9.042107e-06 0.004912448           0.1875             0.7500
##    loss_sample_prop gain_exp_mean normal_exp_mean loss_exp_mean
## 1:           0.0000     0.4967500      -0.2129028           NaN
## 2:           0.0625     2.6232500       0.6852500     -0.670500
## 3:           0.1250    -0.7475000      -1.5413333     -1.505833
## 4:           0.0000     1.2829792       0.4458750           NaN
## 5:           0.1250     0.2345417       1.5736818      2.524063
## 6:           0.0625     1.5993333       0.6277917     -0.069000
##    vs_rest_exp_diff
## 1:        0.7096528
## 2:        2.0509792
## 3:        0.7883718
## 4:        0.8371042
## 5:       -1.4853526
## 6:        1.0251410
```

```
head(cna_driven_genes$loss_driven)
```

```
##      GENE      p_value          fdr gain_sample_prop normal_sample_prop
## 1:   POLK 8.417198e-10 3.447685e-06           0.1250             0.6875
## 2:   EZH1 4.227489e-07 7.088258e-04           0.0000             0.5625
## 3:   SOD2 5.191596e-07 7.088258e-04           0.0625             0.7500
## 4: MRPS36 8.307626e-07 8.245376e-04           0.1250             0.6875
## 5:   NBR1 1.053985e-06 8.245376e-04           0.0625             0.5000
## 6: ZNF554 1.207819e-06 8.245376e-04           0.1250             0.6875
##    loss_sample_prop gain_exp_mean normal_exp_mean loss_exp_mean
## 1:           0.1875     -0.403300      -0.1174404    -1.2971667
## 2:           0.4375           NaN       0.2341667    -0.4589762
## 3:           0.1875      0.066250      -0.2052812    -1.0430417
## 4:           0.1875      0.364375      -0.0490000    -1.0135833
## 5:           0.4375      0.644500       0.8796875    -0.3331786
## 6:           0.1875      0.950625       0.6446591    -0.2225833
##    vs_rest_exp_diff
## 1:       -1.1357479
## 2:       -0.6931429
## 3:       -0.8586474
## 4:       -1.0281795
## 5:       -1.1867341
## 6:       -0.9143141
```

```
head(cna_driven_genes$both)
```

```
##      GENE gain_p_value   gain_fdr loss_p_value   loss_fdr gain_sample_prop
## 1: AHNAK2 0.0001146049 0.01676318  0.103289129 0.32975236           0.1875
## 2:   NTF3 0.0002403053 0.02343285  0.559826577 0.76690624           0.1875
## 3:    ABR 0.0011628790 0.05807826  0.001161531 0.04115464           0.1875
## 4:   RFX3 0.0011221713 0.05807826  0.107495688 0.33610865           0.1875
## 5:  IDH3B 0.0014698661 0.06051011  0.658967508 0.83357965           0.1875
## 6:   FNTB 0.0016109439 0.06315896  0.063966171 0.26626569           0.1875
##    normal_sample_prop loss_sample_prop gain_exp_mean normal_exp_mean
## 1:              0.625           0.1875     0.8897222     -0.26045227
## 2:              0.625           0.1875    -2.8278333     -1.33816667
## 3:              0.375           0.4375     1.0030000      0.64137500
## 4:              0.625           0.1875     0.7407222     -0.05018333
## 5:              0.625           0.1875    -0.5640417     -0.98650000
## 6:              0.625           0.1875     0.7234333      0.33618000
##    loss_exp_mean gain_vs_rest_exp_diff loss_vs_exp_diff
## 1:   -1.08888889             1.3413522       -1.0938615
## 2:   -1.20477778            -1.5204487        0.4771581
## 3:   -0.02914286             0.7226731       -0.7910595
## 4:   -1.00261111             1.0106966       -1.1349444
## 5:   -1.03441667             0.4335160       -0.1454071
## 6:   -0.18523333             0.5075795       -0.6107795
```

For example, BRCA1 appears to be CNA loss-driven in this dataset, and its expression is lower in samples of CNA loss than samples of CNA neutral.

```
cna_driven_genes$loss_driven[GENE %in% c('BRCA1')]
```

```
##     GENE   p_value       fdr gain_sample_prop normal_sample_prop
## 1: BRCA1 0.3259034 0.5930255                0             0.5625
##    loss_sample_prop gain_exp_mean normal_exp_mean loss_exp_mean
## 1:           0.4375           NaN       -1.547074     -1.773119
##    vs_rest_exp_diff
## 1:        -0.226045
```

## Q and As

#### Q: Why required to use the bundled hg19 human genome reference?

In the early phase of development, iGC required a special data structure for genome reference hence one is bundled. Now no such special structure is required, so we plan to relax such constraint in the coming-up release and user will be able to pass in other references available on Bioconductor.

Currently the modified `hg19DBNM` contains RefSeq transcripts of hg19 from UCSC Genome Browser. The transcripts with NM marker ID and protein coding, were selected.
